# Case Report: Combined Preperitoneal Enhanced-View Totally Extraperitoneal (PeTEP) Repair with Intraoperative Fascial Traction after Prehabilitation with Botulinum Toxin A in a Large Congenital Umbilical Hernia

**DOI:** 10.3389/jaws.2025.15056

**Published:** 2025-09-25

**Authors:** Carlos Bustamante-Recuenco, Aritz Equisoain-Azcona, Javier García-Quijada García, Ramón Sanz-Ongil, Sergio Salido-Fernández, Francisco Javier Angulo Morales

**Affiliations:** ^1^ Hospital Central de la Cruz Roja San José y Santa Adela, Madrid, Spain; ^2^ Universidad Alfonso X el Sabio, Villanueva de la Cañada, Spain; ^3^ Hospital Universitario La Paz, Madrid, Spain

**Keywords:** minimally invasive surgery, abdominal wall surgery, umbilical hernia, botulinum toxin A, prehabilitation, intraoperative fascial traction, PeTEP approach

## Abstract

**Introduction:**

Congenital umbilical hernia affects 10% of infants. While 80% of cases resolve spontaneously in early childhood, surgical treatment in adults poses challenges due to progressive growth presented over time. Minimally invasive approaches have gained prominence over the past two decades in abdominal wall surgery, with PeTEP (Preperitoneal Enhanced-View Totally Extraperitoneal) being the latest surgical technique introduced. However, its effectiveness in repairing large hernias remains unverified. In this regard, intraoperative fascial traction (IFT) could facilitate fascial closure and potentially expand the indications of this novel surgical technique.

**Material and Methods:**

A 29-year-old male with arterial hypertension, a BMI of 29 and no prior surgical history was referred for surgical management of a congenital umbilical hernia. He presented with discomfort at the site of the umbilical hernia, exacerbated by movement. Preoperative CT scan revealed an 8.5 cm × 6 cm hernia defect (large-sized according to EHS guidelines) associated with a 10,1 cm rectus diastasis. Prehabilitation with botulinum toxin (BTA) injection followed by PeTEP surgical repair was performed. IFT was succesfully used to assist in the closure of the hernia defect.

**Results:**

Early postoperative recovery was favorable, with the patient experiencing low pain levels and being discharged within a day. A 6 cm asymptomatic seroma was observed 1 month postoperatively and was effectively resolved through conservative management. By the 3-month follow-up, the patient reported full functional recovery with no signs of recurrence and satisfactory cosmetic results.

**Conclusion:**

This case report demonstrates that the PeTEP approach, complemented by BTA prehabilitation and intraoperative fascial traction, is viable for the repair of larger midline hernias. This combined method may enhance functional outcomes and recovery speed. However, additional research is needed to evaluate its long-term effectiveness.

## Introduction

Umbilical hernia appears in 10% of infants, and it is more prevalent in preterm neonates, those with low birth weight, or in case of congenital hypothyroidism or Down syndrome. As it corrects spontaneously during the first years of life in up to 80% of the cases, its treatment is usually reported in the pediatric surgery field [1].

Surgical techniques in abdominal wall field are numerous and live in constant evolution. In this regard, minimally invasive approach has experienced an exponential growth over the past 10 years [2]. In this period we have witnessed a change from the initial intraperitoneal techniques, like the IPOM, IPOM+ and LIRA [3, 4]; to an endoscopic and more anatomical approach with the eTEP and the eTEP-TAR [5]. The benefits of endoscopic surgery seem to be evident when compared to laparoscopic ones, as lower postoperative pain is reported in certain studies, with no significant difference in the rate of other complications [6, 7]. Along these lines, Preperitoneal Enhanced-View Totally Extraperitoneal (PeTEP) is the most recent technique, for its first description was made by Valenzuela et al. in 2024 [8, 9]. The combination of an endoscopic extraperitoneal access with a preperitoneal hernia repair allows for a complete preservation of the musculoaponeurotic complex, with all the advantages this entails. However, its utility in medium-to-large abdominal wall hernias remain to be proven, as well as its long-term outcomes, as no evidence of late recurrence, pain and bulging rate is available at this point.

Parallel to this progress, methods to avoid component separation have been developed. Abdominal wall prehabilitation with botulinum toxin A (BTA) injection, as well as progressive pneumoperitoneum (PPP) have proved to facilitate primary fascial closure without adding significant morbidity [10]. Recently, intraoperative fascial traction has arised as a surgical option to manage large hernia defects with or without loss of domain. Promising results have been obtained both in experimental and clinical settings, even though long-term outcome evidence is still scarce [11, 12].

The performance of PeTEP hernia repair combined with IFT in a patient prehabilitated through BTA injection has not been described in the literature up to now. This case report, in accordance to CARE guidelines [13], describes the initial experience of this mixed surgical approach in a patient with a large congenital umbilical hernia.

## Materials and Methods

### Case Description

A 29-year-old male, natural from Mali, with no previous abdominal surgeries, hypertension as unique morbidity and no known toxic habits was referred to our clinic. He presented with a congenital umbilical hernia that had progressively enlarged over the years, causing significant discomfort and interference with daily work activities. On physical examination, he had a large, reductible umbilical hernia without significant excess skin, and a BMI of 29. Neither excess panniculus tissue nor stretch marks were present.

**FIGURE 1:**
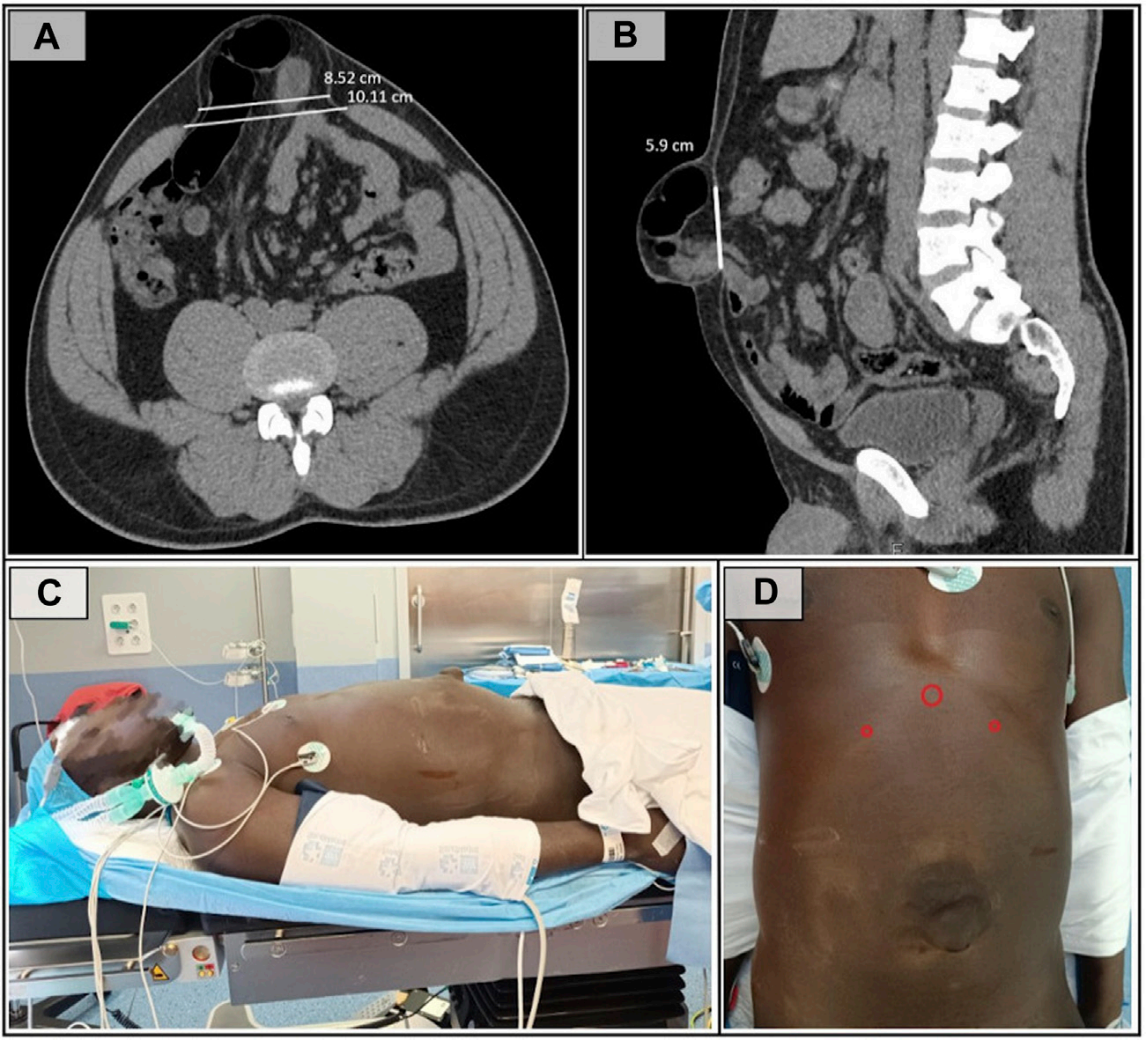
**(A)** Preoperative CT cross-sectional image showing a defect with a transverse diameter of 8.5 cm. **(B)** Saggital CT section demonstrating the 6 cm lenght of the defect. **(C)** Patient in the supine position with arms adducted. **(D)** Trocar placement showing a 12 mm subxiphoid port and 5 mm trocars in both hypocondria.

As part of the preoperative preparation, an abdominopelvic CT scan with Valsalva maneuver was performed, revealing, as shown in Figures 1A, B, a large-sized defect (according to the EHS umbilical and epigastric classification), measuring 8.5 cm width × 6 cm length, with an area defect of 40.07 cm^2^ associated with a severe supraumbilical rectus diastasis (according to EHS rectus diastasis classification) [14] of 10.1 cm.

Given the patient’s young age and physically demanding occupation in the construction industry, a minimally invasive approach was selected. A preperitoneal extended-view totally extraperitoneal (eTEP) hernia repair via a superior access was therefore indicated. This technique offers key advantages, including preservation of the musculoaponeurotic wall and placement of the mesh in the preperitoneal (pretransversalis) space. The patient agreed with the proposed management.

The administration of 300 IU of botulinum toxin (Botox^®^, Allergan Inc., United States) was performed following the Deerenberg technique [15], 6 weeks before the surgical intervention.

### Surgical Technique

The procedure was performed under general anesthesia. A preoperative transversus abdominis plane (TAP) block was completed to minimize postoperative pain. The patient was placed in the supine position with legs and arms adducted. The surgeons were positioned at the head of the patient, while the laparoscopic equipment tower was placed at the foot of the operating table. A slight lumbar extension was applied to enhance the working space and improve ergonomic conditions (Figure 1C).

A video vignette is provided to illustrate the principal steps of the procedure (see https://www.youtube.com/watch?v=rPVN_svN_sc).

#### Preperitoneal Access and Trocar Placement

A 1.5 cm subxiphoid midline incision was made to achieve preperitoneal access. A Hasson trocar was inserted, allowing blunt dissection of the preperitoneal fatty rhomboid, facilitating the placement of two additional 5 mm trocars in both hypochondria (Figure 1D).

#### Pretransversalis Dissection

**FIGURE 2:**
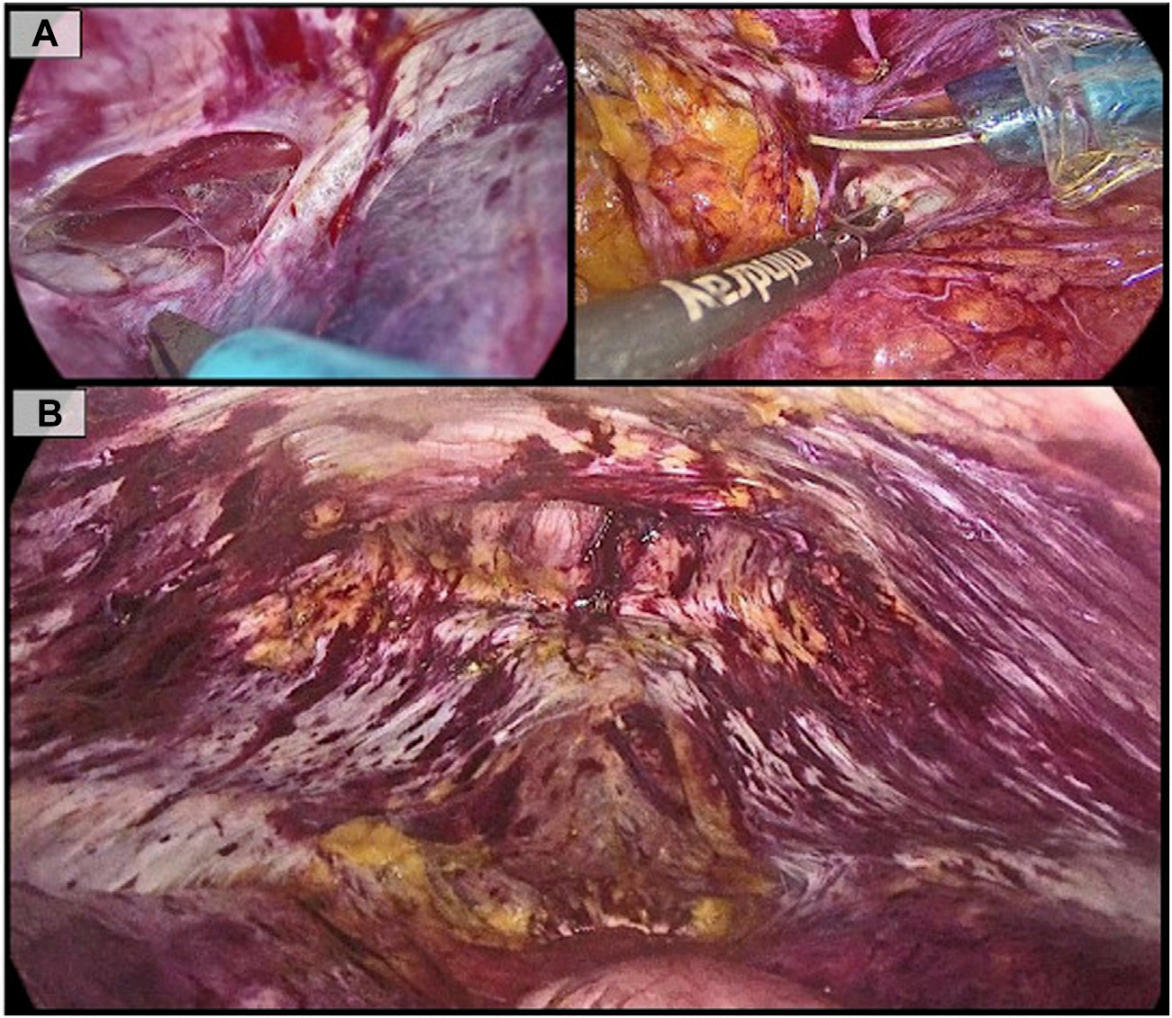
**(A)**: Pre-transversalis access in the left hypochondrium, showing the transversus muscle body. On the contralateral side, division of the transversalis fascia in the right hypochondrium; **(B)**: Final view of the dissected space, showing the hernia defect at the top and both posterior rectus sheaths preserved.

The pretransversalis space was accessed at the hypochondrium by incising the transversalis fascia, exposing the transversus abdominis muscle bilaterally (Figure 2A). Dissection was performed in a laterocaudal direction along the anatomical plane, following the natural insertion of the transversus abdominis into the posterior rectus sheath.

#### Central Preperitoneal Dissection and Hernia Sac Reduction

Only after accessing the pre-transversalis space on both sides, the preperitoneal midline dissection was performed. The hernia sac was only partially reduced, as a significant portion of it was strongly adherent to the skin. Thus, part of the sac was excised and left within the subcutaneous tissue.

After this maneuver, dissection was extended to the retropubic and retroinguinal spaces until the pubic symphysis was visualized (Figure 2B).

#### Midline Reconstruction

**FIGURE 3:**
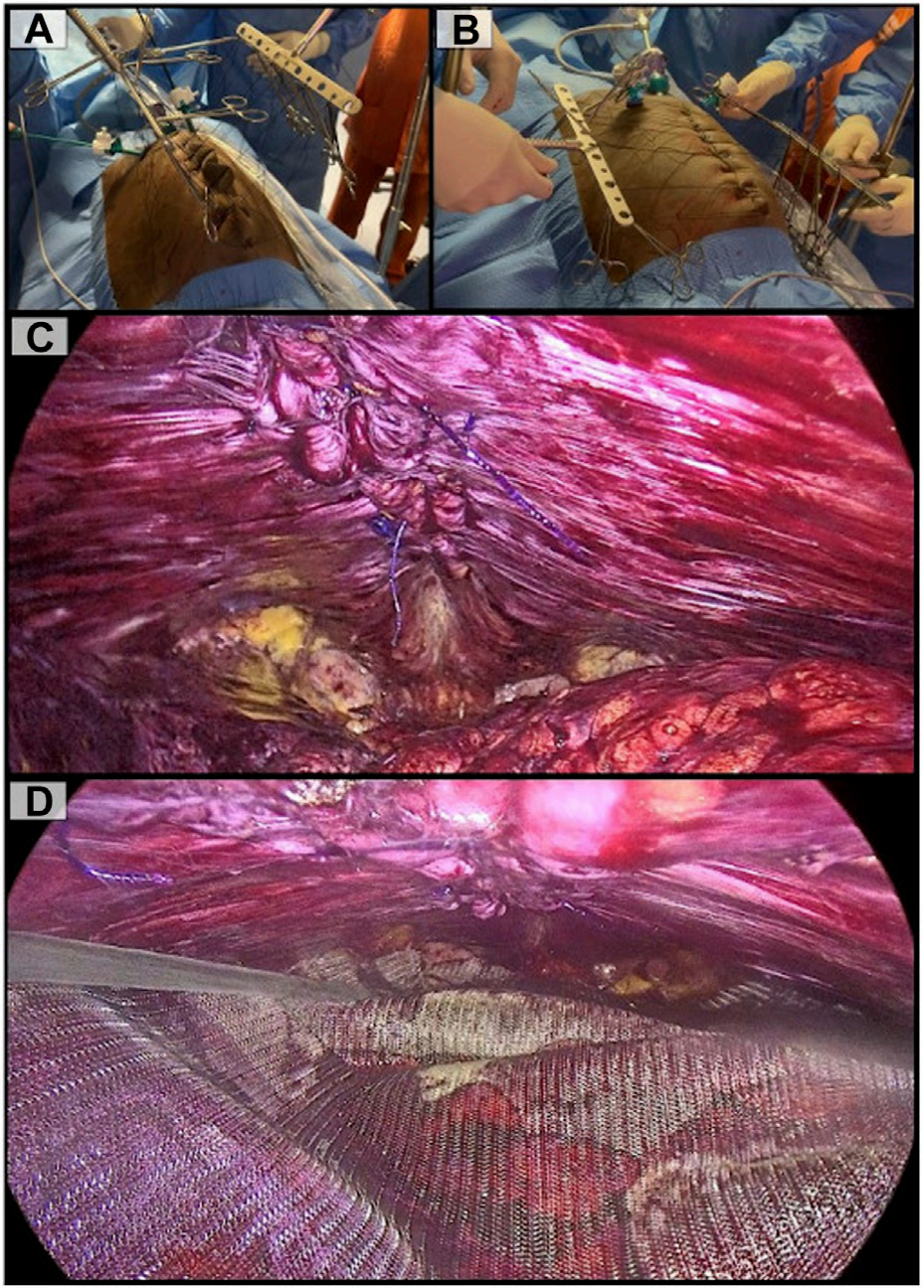
**(A)**: Vertical traction phase aimed at reducing closure tension; **(B)**: Horizontal traction to assess midline reconstruction; **(C)**: Complete plication of the linea alba is performed, closing the defect and restoring normal anatomy; **(D)**: Reinforcement polypropylene mesh is placed in the preperitoneal space.

Hernia defect closure was performed using a fascial traction system (Ansabere^©^, Assut^©^) *due to the significant tension encountered during primary closure, which led to rupture of the suture lines.* Six transfascial sutures (Assufil^©^, Assut Europe^©^) were placed on each side along the medial border of the rectus muscles, exteriorized through the skin, and anchored to the traction system. This novel device consists of a fixed structure that is securely attached to the operating table, onto which two adjustable mechanisms are mounted. These allow repositioning to modify the direction of traction according to the patient’s needs. It also enables the application of progressive traction through the anchored sutures exteriorized through the patient’s skin. The first phase of vertical traction was initiated (Figure 3A), adjusted to the defect size, aiming to elongate the aponeurotic tissue helped by the synergistic effect of botulinum toxin. During the 15 min of vertical traction, peritoneal gaps caused during the dissection were closed.

Subsequently, a second phase of horizontal traction (Figure 3B) was applied immediately prior to the midline reconstruction (hernia defect closure and diastasis plication), and stopped after its completion. This horizontal traction was not sustained for a defined period, but rather employed exclusively as an intraoperative adjunct to facilitate midline closure. Midline reconstruction was performed using a slowly absorbable sized 1 barbed suture with a 37 mm neddle (Filbloc^©^, Assut Europe^©^), as it allowed both midline closure and plication of the hernia sac, thereby reducing the risk of postoperative seroma (Figure 3C).

#### Prosthetic Repair

Finally, a 30 cm × 15 cm (area of 353,43 cm^2^) medium-density polypropylene mesh (Assumesh^©^, Assut Europe^©^) was placed in the preperitoneal/pretransversalis space, without fixation (Figure 3D)

## Results

Early postoperative course was favorable, as the patient exhibited proper oral tolerance, low levels of pain and early mobilization. The patient was discharged the first day after surgery without complications.

**FIGURE 4:**
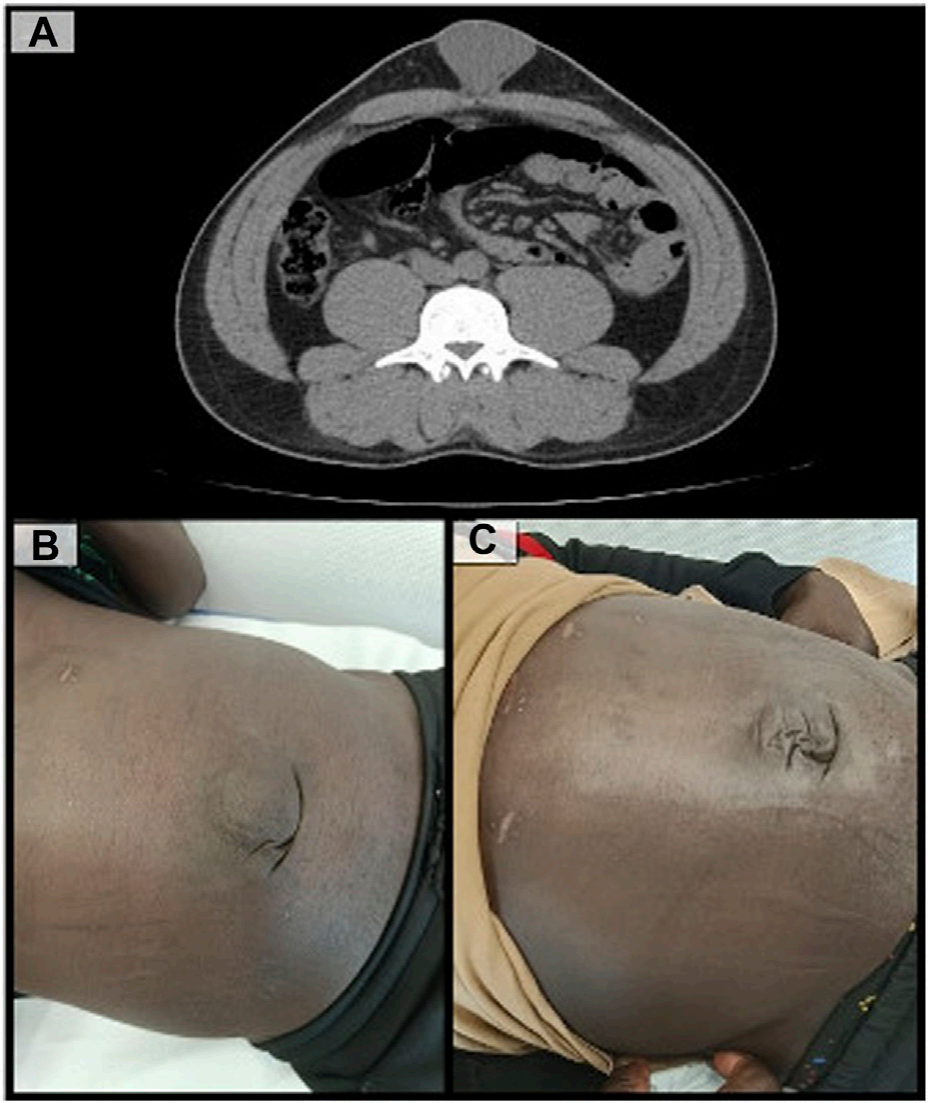
**(A)**: CT scan showing corrected diastasis recti and a significant postoperative seroma; **(B)**: Patient follow-up at one month postoperatively; **(C)**: Patient follow-up at three months postoperatively.

At 1 month postoperatively, the patient developed a 6 cm seroma without other signs of complications (Figure 4A). It was managed conservatively and no other incidents were recorded.

After 3 months of follow-up, the patient achieved full functional recovery, with no objective recurrence, no pain during physical activity and a highly satisfactory cosmetic outcome (Figures 4B, C).

## Discussion

Congenital umbilical hernias appeared due to the lack of obliteration of the umbilical duct, which lead to an incomplete closure of the abdominal wall. They are usually detected and subsequently corrected in the first years of life, but in the absence of proper treatment they can grow over time and reach large sizes, like in the present case [16]. Therefore, its management in adulthood can be challenging, even though general surgical principles of abdominal wall repair can be applied.

The main point to increase the effectiveness of any hernia repair is the primary fascial closure combined with a prosthetic reinforcement in a posterior plane [17]. The first can be difficult or even impossible to achieve in case of large defects without performing an anterior or posterior component separation, an effective procedure that, nevertheless, adds significant morbidity [18]. On the other hand, the preperitoneal space has always been the best theorical plane to place the mesh but the less used through history in midline medium-to-large hernias, being the Rives-Stoppa +/− TAR the gold standard in these cases [17, 19]. Thus, the reach of a complete fascial closure with a posterior mesh reinforcement seems to be the goal to reach.

The search for this ideal technique amidst the ongoing development of minimally invasive surgery has led to the development of the PeTEP. The sole section of the transversalis fascia allows for a complete functional and structural preservation, overcoming the disadvantages of the eTEP [20]. Moreover, the mesh placement in the preperitoneal/pretransversalis plane makes profitable use of the intraperitoneal pressure. Also, the proshtesis can be of great size, as the only dissection limit is the lumbar spine. We chose this technique due to the patient´s youth, its physically demanding profession and the size of the defect. We use a cranial approach, as described by Munoz-Rodriguez et al. for the hernia location and the easier entry to the dissection plane it enables, especially when compared to the suprapubic access [21].

Nonetheless, the dissection of the pre-transversalis space does not allow for a greater approximation of the midline, which constitutes the main limitation of this technique and explains why only small-defect PeTEP series have been reported to date [8, 9, 21, 22]. In our specific case, we decided to use a combination of BTA injection and IFT to solve this issue.

BTA prehabilitation could be consider nearly an standard for EHS W3 hernias up to now [23]. However, its great utility in avoiding component separation is expanding its use to smaller defects. BTA injection causes a relaxation and consecuent elongation of the three muscle layers of the abdominal wall, thus reducing the horizontal hernia diameter in aproximately 4.2 cm, as shown previously by Elstner et al. [24]. This procedure significantly aids in achieving primary fascial closure in large defects, thereby simplifying their treatment.

Even though BTA prehabilitation could have been enough in this case, we decide to combine it with IFT. The hernia size defect, our intention to avoid any component separation and the lack of evidence about the use of botulinum toxin prehabilitation alone in a PeTEP repair leads us to this decision. Also, the excellent clinical results presented by Köckerling with the combination of these two methods provided us the necessary evidence to apply it in this case [25].

IFT is an easy-to-perform and effective technique. No significant complications specific to the technique or device have been reported to date [11, 25]. It can be combined with BTA, CST, and PPP techniques. It is essential to recognise that no single technique can be universally applied to all hernias. Tailoring is key. Each case must be evaluated individually to determine the most suitable approach, utilizing one or a combination of the aforementioned techniques to optimise patient outcomes.

In this specific patient, we put the scope in avoiding any disruption of the musculoaponeurotic complex, which constitutes the main strength of PeTEP. Nevertheless, the use of BTA plus IFT was necessary to achieve the closure, as the application of this technique in ≥8 cm defects is challenging and not proven yet, and is its main limitation at the present moment. ETEP + TAR and PeTEP with endoscopic anterior component separation (eACS) were also considered but both were dismissed to avoid the risk of postoperatory bulging and a possible decrease in the functionality of the abdominal wall [20, 26].

A 6 cm seroma, defined as type II according the Morales-Conde classification, was the only complication recorded [27]. Given the size of the hernia sac and its incomplete reduction, this outcome was anticipated by the team. Nevertheless, just a medium, not limiting discomfort during the usual physical activity was referred by the patient during the first month, and spontaneous resolution was achieved shortly after the first clinical review.

The main limitation of this case report, aside from the inherent methodological ones, is the 3 month follow-up period. Thus, this single result cannot be generalized and further investigation is required to established the long-term outcome and clearly define the indications of this novel surgical method. Also, it is essential to understand that each case has specific characteristics, and no single technique is applicable to all hernias. The scope of abdominal wall surgery, encompassing the preoperative, intraoperative, and postoperative stages, offers surgeons a vast array of options. Consequently, tailoring approaches constitutes the cornerstone of both the present and future of hernia treatment.

## Conclusion

As shown in this report, the preperitoneal approach can be used not only to repair W1-2 hernias, but also in case of larger defects. Moreover, therapeutic tools as BTA prehabilitation and intraoperative fascial traction can be associated in order to optimize postoperatory results. The use of this combined approach in selected cases could enable the treatment of large defects while preserving the myofascial complex of the abdominal wall, leading to improved functional outcomes and faster recovery.

Nevertheless, further investigation is essential to determine the long-term effectiveness and benefits of both PeTEP repair as well as intraoperative fascial traction.

## Data Availability

The original contributions presented in the study are included in the article/Supplementary Material, further inquiries can be directed to the corresponding author.
